# Can Large Language Models Draft Safe, Reliable Patient Leaflets on Driving After Stroke in Comparison to the Stroke Association Leaflet?

**DOI:** 10.7759/cureus.97155

**Published:** 2025-11-18

**Authors:** Mohammed Suleman, Bnar Massraf, Radim Licenik

**Affiliations:** 1 Department of Stroke Medicine, Acute Stroke Centre, North West Anglia NHS Foundation Trust, Peterborough, GBR; 2 Department of Stroke Medicine, Cambridge University Hospitals NHS Foundation Trust, Cambridge, GBR

**Keywords:** artificial intelligence, discern, driver and vehicle licensing agency (dvla), health literacy, large language models, patient education, readability, stroke

## Abstract

Background: Readable and reliable patient information leaflets (PILs) are essential to ensuring patients understand post-stroke driving regulations. With the rapid advancement of large language models (LLMs), there is increasing interest in whether AI-generated materials can achieve quality comparable to expert-authored leaflets such as those produced by the Stroke Association.

Objective: This study aimed to evaluate the readability, reliability, actionability, and factual accuracy of eight "Driving After Stroke" leaflets, seven generated by leading LLMs and one gold-standard version from the Stroke Association, using validated appraisal tools.

Methods: Eight PILs were assessed: the official Stroke Association leaflet and seven AI-generated versions from ChatGPT-5 (Thinking, OpenAI, San Francisco, CA), ChatGPT-5o (Standard, OpenAI), Microsoft Copilot (Microsoft Corp., Redmond, WA), Claude Sonnet 4 (Anthropic, San Francisco, CA), Gemini 2.5 Flash (Google, Mountain View, CA), Perplexity AI (free, Perplexity AI, Inc., San Francisco, CA), and DeepSeek v3.1 (DeepSeek, Hangzhou, China). Each leaflet was converted to plain text for uniform analysis. Readability (Flesch-Kincaid Grade (FKG), Flesch Reading Ease (FRE), Automated Readability Index (ARI), and Gunning Fog Index (GFI)), reliability (DISCERN), understandability and actionability (Patient Education Materials Assessment Tool for Print Materials (PEMAT-P; understandability (U)/actionability (A)), misinformation (0-6; lower = better), and referencing quality (0-2) were scored and synthesised into a weighted composite, adapted from a prior ophthalmology study of AI-generated patient leaflets.

Results: The Stroke Association leaflet achieved the highest composite score (72.8%), followed by DeepSeek v3.1 (68.3%) and Microsoft Copilot (66.4%). ChatGPT-5 (Thinking) ranked closely behind (66.3%), while Gemini 2.5 Flash, ChatGPT-5o (Standard), Claude Sonnet 4, and Perplexity AI (free) scored lower (range 29.8-59.1%). Although no AI model exceeded the gold standard, DeepSeek produced near-comparable readability and structure. Across all leaflets, readability remained within a secondary-school band (median FKG 8.5), with AI-generated texts generally clearer but less well-referenced. The Stroke Association leaflet achieved perfect factual accuracy and referencing, whereas Microsoft Copilot and DeepSeek demonstrated occasional omissions in seizure and transient ischemic attack (TIA) guidance.

Conclusion: Expert-authored materials remain the benchmark for reliability, factual precision, and source transparency. However, newer LLMs such as DeepSeek v3.1 and Microsoft Copilot generated patient leaflets that approached professional standards in readability and usability. These findings support a hybrid approach, AI-assisted drafting with clinician and legal oversight is the most efficient and safe model for future patient education.

## Introduction

Stroke can be a life-changing diagnosis, and for many patients, the complexities of management extend far beyond the acute admission into the post-discharge phase. To help preserve patient autonomy, a range of educational leaflets has been developed to guide this transition, addressing the holistic needs of stroke survivors. Among these, driving after stroke remains a particularly significant topic, often representing a key marker of independence and community reintegration [1-3].

It follows that any authoritative source of information on post-stroke driving must not only convey accurate guidance but also do so in a manner that is clear, accessible, and aligned with the reading level of the average UK citizen. Health literacy, therefore, becomes an independent yet critical determinant of outcomes in stroke recovery, influencing adherence, safety, and patient confidence [4-6]. The subject of driving after stroke is inherently nuanced, encompassing both legal obligations and ethical imperatives linked to patient and public welfare [7,8].

In this context, large language models (LLMs) offer a promising avenue for producing structured, contextually relevant, and readable health information. Their ability to synthesise complex data into succinct, patient-friendly language presents an opportunity to supplement or streamline conventional health communication materials. Yet the reliability of such automatically generated text, particularly where public safety and legal implications are concerned, requires rigorous evaluation [9-11].

The central question, then, is whether LLMs can generate patient information leaflets (PILs) that are not only readable but also accurate, safe, and actionable for stroke survivors. While previous studies have explored AI reliability in answering clinical queries, few have directly compared LLM-generated leaflets against established, expert-authored resources using validated appraisal frameworks [9-11]. This study, therefore, undertakes a comparative evaluation of eight leaflets, seven generated by leading LLMs and one published by the Stroke Association, to determine whether AI systems can match or enhance the communication quality of current gold-standard patient information on driving after stroke in the UK [1].

## Materials and methods

Dataset

We conducted a cross-sectional comparative analysis of eight PILs on post-stroke driving in the UK. The reference standard was the Stroke Association “Driving After a Stroke” leaflet (July 2025 edition) [1]. Seven further leaflets were generated using LLMs: ChatGPT-5 (Thinking, OpenAI, San Francisco, CA), ChatGPT-5o (Standard, OpenAI), Microsoft Copilot (Microsoft Corp., Redmond, WA), Claude Sonnet 4 (Anthropic, San Francisco, CA), Gemini 2.5 Flash (Google, Mountain View, CA), Perplexity AI (free, Perplexity AI, Inc., San Francisco, CA), and DeepSeek v3.1 (DeepSeek, Hangzhou, China). All LLM outputs were produced on 27 September 2025 using the most current publicly available versions at that time. ChatGPT-5 (Thinking) and ChatGPT-5o (Standard) were accessed via a ChatGPT Plus subscription (OpenAI); Microsoft Copilot via the free web interface (copilot.microsoft.com); and DeepSeek v3.1, Gemini 2.5 Flash, Claude Sonnet 4, and Perplexity AI (free) via their respective free public interfaces. Each model received the identical prompt: “Make a patient information leaflet about driving after a stroke in the UK.”

The full text of all seven LLM-generated leaflets is reproduced verbatim in Appendix A (raw corpus) for transparency; the standardised corpus of these seven LLM leaflets used for analysis appears in Appendix B. The Stroke Association leaflet [1] was standardised using the identical protocol (Appendix C); the source document is publicly accessible at the cited URL. All study materials, including raw outputs, standardised texts, and analysis protocols (Appendices A-C), are permanently archived on Zenodo for reproducibility (DOI: 10.5281/zenodo.17496584).

Text standardisation

To ensure methodological consistency and remove potential formatting bias, all documents were converted to plain text prior to analysis. We then applied a uniform, structure-only standardisation: we inserted full stops at the end of bullet points so list items were parsed as complete sentences and rendered items as left-aligned lines (removing visible bullet/hyphen markers); we retained meaningful subheadings to preserve document structure and added a trailing colon if a heading lacked punctuation, and where a heading was interrogative we ensured it ended with a question mark (without an additional colon); and where tabulated material appeared, we flattened tables by concatenating header-cell pairs into short, complete sentences with minimal or no connectives using a punctuation-only label-value pattern (e.g., “Label: value.”) so linguistic software would parse them consistently. We removed citations and references (inline or terminal) and emojis or decorative symbols, normalised whitespace (single spaces, one blank line between sections), and resolved obvious extraction artefacts (e.g., line-break hyphenation). No paraphrasing or lexical edits were made, and original wording, spelling, numerals, abbreviations, and order were preserved. These steps ensured a uniform, analysis-ready format across all subsequent evaluation domains. The complete standardisation protocol is detailed in Appendix C; the standardised corpus is provided in Appendix B, and the raw unstandardised outputs in Appendix A for comparison. While this text-only standardisation protocol controlled for formatting bias across all outputs, it may modestly under- or overestimate Patient Education Materials Assessment Tool for Print Materials (PEMAT-P) items that depend on visual layout features (e.g., spacing, typography, or graphical cues). This was a deliberate trade-off to enable fair textual comparison at the expense of layout-dependent accessibility.

Evaluation framework

We assessed five complementary domains to capture linguistic accessibility, reliability, and patient-centred quality: readability, reliability (DISCERN), understandability and actionability (PEMAT-P; U/A), misinformation, and referencing quality [12,13,14-17].

Readability

Readability was quantified using four established indices: Flesch-Kincaid Grade (FKG), Flesch Reading Ease (FRE), Automated Readability Index (ARI), and Gunning Fog Index (GFI) [14-17]. Briefly, FKG estimates the U.S. school grade level required for comprehension [15]; FRE ranges from 0 to 100, with higher scores indicating easier readability [14]; ARI reflects difficulty based on characters per word and words per sentence [16]; and GFI estimates years of formal education needed for understanding [17]. Indices were computed after text normalisation (Readability Formulas, readabilityformulas.com). Each readability index was weighted equally (1.0) in the composite to avoid over-privileging any single construct.

Reliability

The DISCERN instrument (score range 16-80) was used to evaluate reliability and overall quality of patient information, focusing on transparency, balance, and evidence support [13]. Scores were independently assigned and cross-checked against current Driver and Vehicle Licensing Agency (DVLA) and Stroke Association guidance to ensure clinical accuracy and UK-specific context [1,7].

Understandability and Actionability

The PEMAT-P was applied in two domains: PEMAT-U (clarity, organisation, navigation) and PEMAT-A (support for concrete next steps) [12]. Both were expressed as percentages. To avoid overemphasising layout over content, PEMAT-U and PEMAT-A were weighted equally at 0.75 each (1.5 combined) in the composite.

Misinformation

We applied a bespoke six-point misinformation framework adapted from prior digital-health evaluations [9,10,11]. Each leaflet was assessed across three subdomains, namely, (i) inappropriate content, (ii) missing content, and (iii) potential harm, scored 0-2 per subdomain (0 = none detected; 1 = minor ambiguity/omission without safety implications; 2 = major omission or phrasing with potential safety consequences). The rubric is provided in Table 1, and item-level rationales are presented in the Results.

**Table 1:** Misinformation scoring framework Each subdomain scored  0-2, with total scores ranging 0-6 (lower = better quality)

Subdomain	Score = 0	Score = 1	Score = 2
Inappropriate content	No misleading or unsafe phrasing	Minor ambiguous phrasing without safety impact	Clear misleading phrasing or potentially unsafe advice
Missing content	No omissions of key UK-specific rules	Minor omission that does not change safety	Major omission of key rule/requirement affecting safety/legal status
Potential harm	No statements likely to cause harm	Low likelihood of harm if misinterpreted	Non-trivial risk of harm or legally risky advice

Referencing Quality

Referencing was scored 0-2 according to clarity and traceability: 2 = explicit, verifiable references (e.g., named guidelines or specific URLs); 1 = generic/ambiguous attributions (e.g., “GOV.UK” without a document title); 0 = no referencing.

Scoring procedures and synthesis

All raw domain metrics were normalised to a 0-1 scale using min-max transformation so that higher values consistently indicated better performance. Accordingly, readability indices that increase with difficulty (FKG, ARI, GFI) and the misinformation score were reverse-coded; FRE, DISCERN, PEMAT-U/A, and referencing were normalised directly. Pre-specified weights were applied as follows: FKG (1.0), FRE (1.0), ARI (1.0), GFI (1.0), DISCERN (1.5), PEMAT-U (0.75), PEMAT-A (0.75), Misinformation (2.0), and References (0.5). The composite score was the weighted sum of normalised metrics; the formula is provided below:

$S = \sum (W_i \times N_i)$

where Wᵢ represents the weighting for each domain and Nᵢ the normalised value.

Given the small corpus (n=8), min-max normalisation can be sensitive to extreme values; composite ranks should therefore be interpreted as relative rather than absolute. 

Quality assurance

To ensure consistency in subjective scoring, all DISCERN and PEMAT-P assessments were independently completed by two evaluators (MS, BM). Disagreements were minor (PEMAT-P ≤10 percentage points; DISCERN ≤5 points) and were resolved through structured discussion until consensus. An independent consultant stroke physician (RL) performed spot checks across all AI-generated leaflets; no major factual inaccuracies were detected, though minor omissions were noted and factored into misinformation scoring. All text standardisation protocols were applied uniformly across leaflets.

## Results

Eight patient information leaflets were analysed, seven generated by LLMs and one expert, authored leaflet from the Stroke Association (July 2025 edition) [1]. Each leaflet was evaluated across readability, reliability, understandability, actionability, misinformation, and referencing domains.

Table 2 presents the raw domain scores for all evaluated leaflets prior to weighting and normalisation. These values form the basis for the composite analysis described below.

**Table 2:** Raw evaluation data for all leaflets across readability indices, reliability (DISCERN), understandability and actionability (PEMAT-P; U/A), misinformation score and referencing quality. Note: For FKG, ARI, Gunning Fog, and Misinformation, lower scores indicate better performance; for all other metrics, higher scores indicate better performance. FKG: Flesch-Kincaid Grade; FRE: Flesch Reading Ease; ARI: Automated Readability Index; PEMAT-P: Patient Education Materials Assessment Tool for Print Materials; PEMAT-U: Patient Education Materials Assessment Tool- Understandability; PEMAT-A: Patient Education Materials Assessment Tool-Actionability

Source	Word Count	FKG	FRE	ARI	Gunning Fog	DISCERN (16–80)	PEMAT-U (%)	PEMAT-A (%)	Misinformation (0–6)	References (0–2)
Stroke Association	1185	8.3	60	8.63	9.7	65	76.9	40	0	2
ChatGPT-5 (Thinking)	532	7.94	63	9.06	10.3	58	76.9	60	1	1
ChatGPT-5o (Standard)	1346	9.71	48	10.78	11.4	64	84.6	60	0	0
Microsoft Copilot	380	6.39	45	6.93	7.8	39	92	60	2	1
DeepSeek v3.1	790	7.5	68	8.48	9.2	48	84.6	60	2	1
Gemini 2.5 Flash	946	8.64	56	9.09	10.3	55	76.9	40	1	1
Claude Sonnet 4	850	9	44	9.83	11.9	59	92	60	2	0
Perplexity AI (free)	453	10.53	48	11.82	11.8	38	76.9	40	3	1

**Figure 1:**
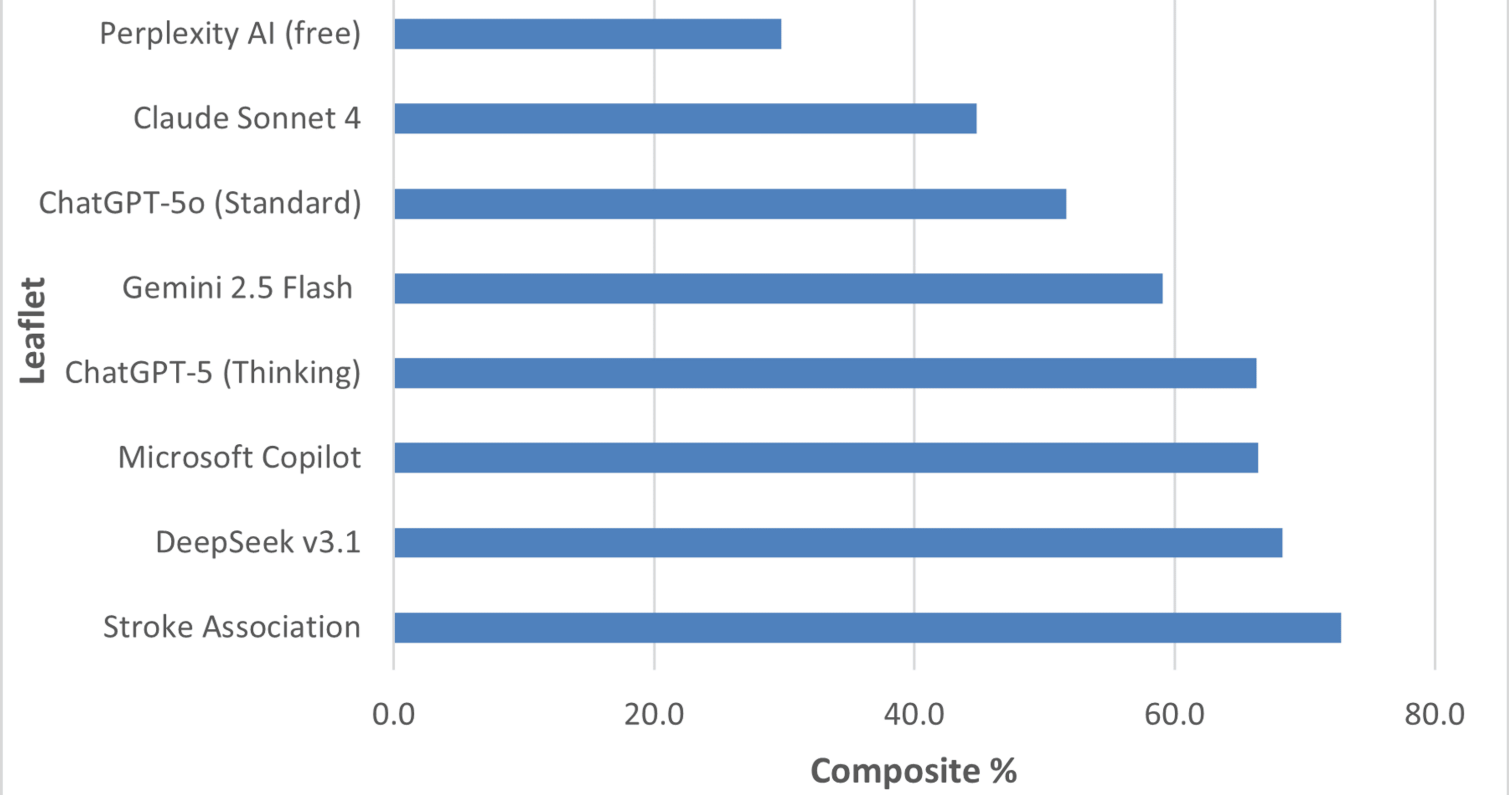
Weighted composite performance scores (%) for all eight patient information leaflets on driving after stroke. Scores represent normalised, weighted metrics across readability, reliability (DISCERN), understandability/actionability (PEMAT), misinformation, and referencing domains. The Stroke Association expert leaflet scored highest (72.8%), followed by DeepSeek v3.1 (68.3%) and Microsoft Copilot (66.4%). PEMAT: Patient Education Materials Assessment Tool

To enable direct comparison across domains of differing scales, scores were normalised and weighted according to the framework outlined in the Methods section. The resulting weighted composite performance for each leaflet is illustrated in Figure 1, showing overall ranking by total percentage score.

Following this, Table 3 provides the corresponding percentage values used in the composite calculation, alongside relative ranking order and key domain highlights for interpretive clarity.

**Table 3:** Ranked performance with key qualitative observations Full domain-by-domain analysis is presented in Results. FKG: Flesch-Kincaid Grade; FRE: Flesch Reading Ease; ARI: Automated Readability Index; TIA: transient ischemic attack; PEMAT-P: Patient Education Materials Assessment Tool for Print Materials; PEMAT-U: Patient Education Materials Assessment Tool-Understandability

Ranking	LLM/Source	Composite Score (%)	Brief Qualitative Performance Summary
1	Stroke Association	72.8%	Most balanced overall. Excellent reliability, zero misinformation, full referencing. Slightly higher reading grade but best safety and traceability.
2	DeepSeek v3.1	68.3%	Strong readability and structure (FRE 68.0, PEMAT-U 84.6). Minor omissions on seizure/TIA detail. Reliable but not fully evidenced.
3	Microsoft Copilot	66.4%	Simplest prose (lowest FKG/ARI). Excellent readability and layout (PEMAT 92%), but lowest reliability (39/80) and moderate misinformation.
4	ChatGPT-5 (Thinking)	66.3%	Balanced readability and factual accuracy. Moderate reliability (58), minor omissions; partial references.
5	Gemini 2.5 Flash	59.1%	Clear presentation and moderate reliability. Minor misinformation; concise but occasionally lacking depth.
6	ChatGPT-5o (Standard)	51.7%	Good reliability (64) and 0 misinformation, but lower readability and no referencing; structurally plain.
7	Claude Sonnet 4	44.8%	Very strong structure (PEMAT-U 92%), but high reading grade and limited evidence base. Moderate misinformation.
8	Perplexity AI (free)	29.8%	Lowest performer overall. Fragmented structure, multiple factual gaps, weak reliability (38), and inconsistent flow from a retrieval-based design.

Overall performance

Across eight PILs (one expert-authored; seven LLM-generated), the Stroke Association leaflet achieved the highest composite score (72.8%), followed by DeepSeek v3.1 (68.3%) and Microsoft Copilot (66.4%) (Figure 1; Table 3). ChatGPT-5 (Thinking) scored 66.3%, Gemini 2.5 Flash 59.1%, ChatGPT-5o (Standard) 51.7%, Claude Sonnet 4 44.8%, and Perplexity AI (free) 29.8%. While no AI model exceeded the gold-standard leaflet, DeepSeek v3.1 trailed the benchmark by only 4.5 percentage points. Because the pre-specified weights prioritised safety-relevant domains (reliability, misinformation, referencing), the expert leaflet maintained its lead despite strong AI performance in readability and structure.

Readability

Readability varied substantially across sources (Table 2). Microsoft Copilot produced the most accessible text by three measures, achieving the lowest FKG (6.39), ARI (6.93), and GFI (7.8). DeepSeek v3.1 recorded the highest FRE (68.0). The Stroke Association leaflet demonstrated balanced readability (FKG 8.30, FRE 60.0, ARI 8.63, GFI 9.7), exceeding the AI median on three of four indices (AI median: FKG 8.64, FRE 48.0, ARI 9.09, GFI 10.3). Several AI outputs showed greater complexity, including ChatGPT-5o (FKG 9.71, FRE 48), Claude Sonnet 4 (FRE 44, GFI 11.9), and Perplexity AI (free) (FKG 10.53, FRE 48). All leaflets remained within a secondary-school-to-early-college readability band.

Reliability (DISCERN)

DISCERN scores (16-80) varied by source (Table 2). The Stroke Association leaflet scored highest (65), followed by ChatGPT-5o (64) and Claude Sonnet 4 (59). Mid-range scores included ChatGPT-5 (Thinking) (58) and Gemini 2.5 Flash (55), while DeepSeek v3.1 scored 48. Microsoft Copilot recorded the lowest DISCERN score (39), reflecting limitations in discussing DVLA reporting pathways, assessment options, transparency, and source attribution despite strong readability. Perplexity AI (free) scored second-lowest (38). This domain represented the clearest separation between expert-authored and AI-generated content.

Understandability and Actionability (PEMAT-U/PEMAT-A)

For structural clarity (PEMAT-U), Microsoft Copilot and Claude Sonnet 4 achieved the highest ratings (92%), with ChatGPT-5o and DeepSeek v3.1 both at 84.6%. The Stroke Association, ChatGPT-5 (Thinking), Gemini 2.5 Flash, and Perplexity AI (free) scored 76.9% (Table 2). For actionability (PEMAT-A), ChatGPT-5 (Thinking), Microsoft Copilot, DeepSeek v3.1, Claude Sonnet 4, and ChatGPT-5o (Standard) scored 60%, whereas the Stroke Association, Gemini 2.5 Flash, and Perplexity AI (free) scored 40%. These patterns indicate that several AI-generated leaflets provided clearer navigational layout and more explicit next-step guidance than the benchmark, addressing domains where patients often struggle most.

Misinformation

On the bespoke misinformation screen (0-6; lower = better), the Stroke Association leaflet and ChatGPT-5o (Standard) scored 0 (no detected omissions or inaccuracies). Minor issues (score 1) appeared in ChatGPT-5 (Thinking) and Gemini 2.5 Flash, typically reflecting omissions of contextual detail rather than explicit inaccuracies.

Moderate concerns (score 2) were identified in Microsoft Copilot, DeepSeek v3.1, and Claude Sonnet 4, often involving ambiguity around seizure waiting periods or incomplete coverage of multiple-TIA scenarios. Perplexity AI (free) scored 3, reflecting both factual gaps and advice that lacked sufficient precision for safety-critical decisions. Table 4 provides a detailed item-level rationale.

**Table 4:** Item-level misinformation assessment with justifications for scores assigned to each leaflet across three subdomains (inappropriate content, missing content, potential harm) DVLA: Driver and Vehicle Licensing Agency; STR1V: Standardised Transport Request 1V; TIA: transient ischemic attack

Source	Inappropriate Content (0–2)	Missing Content (0–2)	Possible Harm (0–2)	Total (0–6)	Brief Rationale
Stroke Association	0	0	0	0	Complete; aligned with DVLA guidance; no issues detected.
ChatGPT-5 (Thinking)	0	1	0	1	Missing psychosocial support.
ChatGPT-5o (Standard)	0	0	0	0	Complete; aligned with DVLA guidance; no issues detected.
Microsoft Copilot	0	2	0	2	Missing STR1V form, multiple-TIA rule, vehicle adaptation, psychosocial support, and consequences of non-compliance. Otherwise, factually correct.
DeepSeek v3.1	1	1	0	2	FED1-APP1 doesn’t exist as a DVLA form
Gemini 2.5 Flash	0	1	0	1	No obvious omissions besides information about psychosocial support.
Claude Sonnet 4	1	1	0	2	Minor overstatement of immediacy in informing DVLA (not required until after four weeks for G1 unless deficits persist). Missing fine/prosecution and DVLA form names. No info about recurrent TIAs within a short span.
Perplexity AI (free)	0	2	1	3	Absence of £1000 fine/prosecution. Absence of DVLA form name. No reference to alternatives to driving. “Don’t inform DVLA in the first month” could mislead. Limited safety emphasis.

Referencing quality

Referencing represented the clearest point of separation between expert-authored and AI-generated content. The Stroke Association leaflet scored 2/2 by providing clear, specific, and verifiable citations to DVLA, NHS, and Stroke Association documents. Among AI models, ChatGPT-5 (Thinking), Microsoft Copilot, DeepSeek v3.1, Gemini 2.5 Flash, and Perplexity AI (free) scored 1/2, typically using generic attributions (e.g., "GOV.UK") without document specificity or section references. ChatGPT-5o (Standard) and Claude Sonnet 4 provided no references (0/2) (Table 2).

## Discussion

Summary of principal findings

In this head-to-head comparison, the expert-authored Stroke Association leaflet retained the highest composite score (72.8%), with DeepSeek v3.1 (68.3%) and Microsoft Copilot (66.4%) ranking second and third. While several LLMs matched or exceeded the benchmark on readability and PEMAT domains (structure and actionability), the expert leaflet prevailed when safety-relevant domains-reliability, misinformation screening, and referencing-were weighted as pre-specified. This outcome aligns with expectations for safety-critical topics in regulated jurisdictions, where source transparency and legal precision are paramount.

Interpretation in context

Readability and Usability Gains From LLMs

Multiple AI outputs achieved excellent readability and high PEMAT scores, addressing real patient needs. Microsoft Copilot produced the simplest prose on three indices (FKG, ARI, and Gunning Fog), while DeepSeek v3.1 achieved the highest reading ease score (FRE 68) while maintaining strong structural clarity (PEMAT-U 84.6%). These features matter clinically: stroke survivors often struggle with "what to do next", and clearer headings, logical chunking, and explicit next-step guidance can improve confidence and adherence to medical advice [2,3,5].

Provenance Determines Trust and Safety

Once reliability, misinformation, and referencing were incorporated into the composite score, the expert leaflet's advantage became clear. The Stroke Association document supplied traceable sources and aligned tightly with UK-specific DVLA guidance, supporting both clinical utility and legal defensibility [1,7]. By contrast, many LLM outputs used generic attributions or omitted details with safety implications, such as multiple-transient ischemic attack (TIA) driving restrictions, seizure waiting periods, or insurance notification requirements.

Predictable Trade-Offs Across Architectural Designs

Models that optimised strongly for linguistic simplicity (e.g., Microsoft Copilot) did not lead to reliability or citations; models that produced more comprehensive text (e.g., Claude Sonnet 4, ChatGPT-5o (Standard)) sometimes increased reading grade or still lacked full provenance. DeepSeek v3.1 balanced readability and structure with moderate reliability (DISCERN 48/80), explaining its second-place finish. Perplexity AI (free), a retrieval-centric system, underperformed across reliability and misinformation domains, reflecting the inherent limitations of aggregating snippets rather than synthesising cohesive, evidence-grounded guidance for safety-critical contexts.

Comparison with prior work

To our knowledge, this represents the first head-to-head evaluation comparing AI-generated and expert-authored patient materials in a stroke-specific, safety-critical context. Our approach extends prior evaluations of AI patient materials by explicitly weighting safety-critical domains alongside readability, providing a more holistic quality assessment [9-11]. Earlier studies have demonstrated that LLMs often match or exceed human-level readability while lagging on evidence transparency and jurisdictional nuance. Using a structured weighting scheme and bespoke misinformation rubric, we localised where specific risks arose, for instance, ambiguous seizure guidance, incomplete multiple-TIA rules, or missing insurance notification advice, complementing global measures such as DISCERN and PEMAT with actionable, granular feedback.

Interestingly, Microsoft Copilot's performance diverged substantially from findings in Massraf et al. [9], where Copilot ranked among the poorest performers (42.7%, second-to-last) with significant reliability and misinformation concerns. Since that study, Microsoft Copilot has undergone major updates; in our evaluation, it demonstrated markedly improved readability (perfect scores on three indices) and strong PEMAT performance (92% understandability). However, reliability limitations persisted (DISCERN 39/80, the lowest among all sources), and misinformation risk remained elevated (score 2/6). This divergence suggests that recent architectural improvements enhanced linguistic fluency and structural clarity, yet medical content reliability and evidence-based reasoning remain areas of concern. The finding reinforces that advances in language generation technology do not automatically translate to domain-specific expertise or the evidential rigour that healthcare communication demands. Comparisons with ophthalmology-focused work reach a similar high-level conclusion: LLMs can generate readable, plausible text; expert review remains essential to secure accuracy, completeness, and source traceability [9].

Practical implications

Several actionable insights emerge from this evaluation.

Do Not Remove Expert Review

Even top-performing LLMs demonstrated gaps in referencing and occasional ambiguity in areas with legal consequences. DeepSeek v3.1's moderate DISCERN score (48/80) and Microsoft Copilot's lower performance (39/80) underscore that no current LLM reliably produces publication-ready materials without verification.

Use LLMs Deliberately

For initial drafting, rapid updating, and multilingual adaptation, reasoning-oriented models like DeepSeek v3.1 and ChatGPT-5 (Thinking) provide useful starting points given their balanced performance across readability and moderate reliability. Productivity-oriented tools like Microsoft Copilot excel at clarity but require reinforcement on evidence transparency and source attribution. Final sign-off requires clinical and legal oversight.

Enforce Provenance Standards

Generic attributions such as "GOV.UK" are insufficient for publication-ready materials. Any AI-assisted leaflet must include specific, checkable sources, such as document titles, section references, and URLs, before dissemination. The Stroke Association's citation of named DVLA guidance documents exemplifies good practice for traceable, named guidance [1].

Verify Jurisdictional Specifics Locally

UK-specific standards, including DVLA reporting procedures (forms STR1/STR1V), Esterman visual field testing criteria, seizure-related waiting periods, multiple-TIA rules, and insurance notification requirements, must be validated by qualified professionals familiar with local regulatory frameworks [1,7]. LLMs cannot reliably infer such nuance without expert verification.

Limitations

Several limitations merit consideration. Subjective scoring is inherent to DISCERN and PEMAT frameworks, though we followed validated rubrics and applied pre-declared weights consistently [12,13]. The dataset (n=8) supports comparative profiling but limits statistical inference. LLM versions evolve continuously; exact replication may vary over time, as evidenced by Microsoft Copilot's performance shift since the Massraf study [9]. Additionally, included LLMs differ in design and default behaviours; despite controlling for prompt, generation date (27 September 2025), and default front-end settings, between-model differences should be interpreted descriptively rather than causally. Normalisation choices (min-max transformation; direction of benefit for each metric) influence rank order; however, these were specified a priori and applied uniformly across all sources. The topic and jurisdiction (UK, post-stroke driving) may limit generalisability to other clinical contexts or regulatory environments. Additionally, because PEMAT-P includes layout-dependent elements, scoring on text-only versions may differ slightly from original formatted documents. Future work with larger corpora could compare alternative scaling methods (e.g., z-scores or percentile ranks) to confirm ranking stability.

Future studies should explore reader-based validation by involving stroke survivors and carers in rating comprehension, trustworthiness, and practical utility directly. Expanding evaluation to multilingual settings or non-stroke conditions could help determine how LLMs handle cross-speciality health communication and cultural adaptation.

Clinical and educational relevance

From a stroke clinician's perspective, the friction is real: patients frequently struggle to interpret DVLA rules, and "what happens if...?" scenarios, such as early post-stroke seizures, multiple TIAs, or visual field loss, are common sources of confusion and anxiety [2,3]. Materials that are both readable and action-oriented reduce patient anxiety and improve adherence to guidance, but they must also be legally correct and citable [5,6]. Well-designed educational materials, whether AI-assisted or expert-authored, serve not only as information tools but also as instruments of reassurance that reinforce safe behaviour and maintain trust in medical advice.

While the gold-standard leaflet maintained superiority across key domains, particularly reliability, misinformation avoidance, and referencing, the narrow margin to DeepSeek v3.1 (4.5 percentage points) suggests LLMs are approaching professional quality in specific contexts. This capability could enable clinical educators and stroke teams to focus on tailoring materials for individual patient needs rather than drafting content from scratch.

The most efficient and safe model for producing patient-facing materials is likely a hybrid, multidisciplinary workflow: LLMs generate clear, well-structured first drafts emphasising accessibility and next-step guidance; clinicians, occupational therapists, and legal advisors then refine content for accuracy, legal compliance, jurisdictional specificity, and source transparency prior to publication. This approach couples technological efficiency with the clinical accountability that underpins patient trust, preserving both speed and safety. As clinicians, our role extends beyond medical decision-making to empowering patients through accessible education. Integrating AI within this process offers a tangible means to achieve efficiency while preserving the human oversight essential to safe, compassionate stroke care.

Looking ahead, as LLMs approach benchmark quality in standardised materials, the next frontier may be personalised patient education AI systems that integrate individual medical histories, comorbidities, and risk profiles to generate truly patient-specific guidance. Such tailored materials could enhance adherence and health outcomes by ensuring that every recommendation reflects the patient's unique clinical context, though this vision will still require robust clinical oversight and regulatory frameworks to ensure safety and accuracy.

## Conclusions

In summary, this study demonstrates that while professionally developed PILs maintain measurable advantages in reliability, accuracy, and source transparency, certain LLMs, particularly DeepSeek v3.1, can generate materials that closely approach gold-standard quality in readability and structural clarity. Microsoft Copilot exemplified the limitations of optimising for linguistic simplicity alone, achieving optimal readability yet scoring lowest in evidential quality. The optimal future model for patient communication will likely combine AI-assisted drafting with expert review, leveraging the efficiency and accessibility of LLMs while ensuring clinical nuance, legal accuracy, and referencing integrity through multidisciplinary human oversight. As these technologies continue to mature, the prospect of truly personalised, patient-specific educational materials, integrating individual medical histories and risk profiles, may further enhance adherence and outcomes, provided such advances remain anchored in clinical accountability and rigorous validation.

## Appendices

Appendix A

Raw LLM Outputs (Verbatim)

The outputs below are reproduced verbatim (content) as generated on 27 September 2025. These verbatim texts represent the seven LLM-generated leaflets only; the Stroke Association leaflet is not reproduced and is cited as reference [1]. Bracketed numbers (e.g., "[1]") and citation markers (e.g., "GOV.UK+2") appear as generated by the models and are not part of this manuscript's reference list. For Gemini 2.5 Flash, original square brackets were converted to full-width brackets〔〕to prevent confusion with this manuscript's references (see Gemini section note). The Cureus editor does not preserve table formatting; where tables appeared in the original output (DeepSeek v3.1 and ChatGPT-5o), we present the text linearly with row separators. Text is unchanged; only visual layout differs. The original formatted outputs are permanently archived at DOI: 10.5281/zenodo.17496584. These differ from the standardised versions used for analysis (see Appendix B).

Perplexity AI (free) - Raw Output

Driving After Stroke: Patient Information Leaflet for Adults in the UK

Important Safety Information

Do not drive for at least 1 month following a stroke or transient ischemic attack (TIA or mini-stroke).

For commercial drivers (e.g., lorry, bus), the minimum driving ban is 1 year.

You do not need to inform the Driver and Vehicle Licensing Agency (DVLA) within the first month, but after one month, you must inform them if you have ongoing effects from your stroke that could affect driving.

Effects That Must Be Reported to DVLA After One Month

Notify the DVLA if you have any of the following ongoing symptoms affecting your driving ability:

Weakness or lack of coordination in arms or legs

Vision problems such as visual field loss, double vision, or visual neglect

Problems with memory, understanding, concentration, or slower reaction times

Ongoing tiredness or cognitive problems

Seizures after the stroke (except for seizures within 24 hours of the stroke)

Brain surgery related to stroke treatment

Multiple strokes within the past three months

Any professional medical concerns about your driving ability

If you hold a Group 2 licence (professional drivers)

Telling Your Insurance Company

It is essential to inform your insurance company that you have had a stroke or TIA. Failure to do so may make your insurance invalid.

Returning to Driving

After the initial 1-month period, discuss with your GP or healthcare professional whether you are fit to resume driving.

You may be referred for a driving assessment to evaluate your fitness to drive safely.

Driving assessments can also be self-referred and help determine if vehicle adaptations or further rehabilitation are needed.

Vision After Stroke

Visit an optician to check your vision if you have concerns post-stroke.

Some visual impairments may require formal assessments before returning to driving.

Additional Notes

If you have had multiple TIAs, you must not drive for 1 month after each episode.

For seizures related to stroke, specific rules apply, including driving restrictions for 6 to 12 months depending on the seizure type.

If you experience cognitive or language difficulties such as aphasia, speak to your healthcare provider for guidance on driving safety.

Support and Resources

Driving Mobility and local driving assessment centres offer specialised assessments and advice.

Speak to stroke support services for additional help navigating your return to driving.

This leaflet aims to support safe and responsible driving after a stroke while complying with UK legal and medical regulations. Always consult your healthcare provider for personalised advice.

If needed, a printable version of official guidance is available from NHS and stroke charity websites.

This information was compiled from UK stroke services, the DVLA medical fitness to drive rules, and stroke charity advice as of September 2025.

Claude Sonnet 4 - Raw Output

Driving After Stroke

Patient Information Leaflet

Understanding Your Situation

Having a stroke can be life-changing, and you may be wondering when or if you'll be able to drive again. This leaflet provides essential information about returning to driving safely and legally after a stroke.

The good news: Many people do return to driving after stroke, but it's important to follow the proper steps to ensure your safety and that of other road users.

Immediate Action Required

You must stop driving immediately after a stroke. This is both a legal requirement and essential for safety. You cannot drive for at least one month after a stroke, and in many cases longer.

Legal Requirements in the UK

DVLA Notification

You must inform the Driver and Vehicle Licensing Agency (DVLA) about your stroke

This must be done as soon as possible after your stroke

Failure to inform DVLA is a criminal offence

Contact DVLA on 0300 790 6806 or visit gov.uk/stroke-and-driving

Minimum Waiting Periods

Car or motorcycle licence: Cannot drive for at least 1 month after stroke

Bus, coach, or lorry licence: Cannot drive for at least 12 months after stroke

These are minimum periods - you may need to wait longer depending on your recovery

Medical Assessment

Before you can return to driving, you need:

Healthcare Team Clearance

Your doctor, occupational therapist, or stroke specialist must assess your fitness to drive

They will evaluate your physical abilities, vision, cognitive function, and reaction times

You may need multiple assessments as you recover

Possible Assessments Include:

Physical examination of strength, coordination, and mobility

Vision tests (including visual field testing)

Cognitive assessments (memory, concentration, decision-making)

Reaction time testing

On-road driving assessment with a specialist instructor

Common Stroke Effects That May Affect Driving

Physical Effects:

Weakness or paralysis on one side of the body

Reduced coordination and balance

Fatigue and reduced stamina

Vision Problems:

Loss of visual field (hemianopia)

Double vision

Difficulty judging distances

Cognitive Effects:

Problems with concentration and attention

Memory difficulties

Slower processing of information

Difficulty making quick decisions

Communication:

Speech problems don't necessarily prevent driving, but severe difficulties may affect safety

Vehicle Adaptations

If you have ongoing difficulties, your car may be adapted to help you drive safely:

Common Adaptations:

Hand controls for acceleration and braking

Steering wheel knobs or handles

Left foot accelerator pedals

Mirrors to compensate for visual field loss

Seat modifications for better positioning

Getting Adaptations:

Speak to an occupational therapist or driving assessment specialist

Some adaptations may be funded through local authority grants or charities

All adaptations must be approved and properly fitted

Insurance Considerations

Informing Your Insurer:

You must tell your car insurance company about your stroke

This should be done immediately, even before you return to driving

Your premium may change, but driving without disclosure could invalidate your policy

Shop around as some insurers specialise in covering drivers with medical conditions

The Return to Driving Process

Step 1: Receive medical clearance from your healthcare team Step 2: Get approval from DVLA (they may request medical reports) Step 3: Inform your insurance company Step 4: Consider a driving assessment with a specialist instructor Step 5: Start with short, familiar journeys in good conditions Step 6: Gradually build up your driving as confidence and ability improve

Warning Signs to Stop Driving

Stop driving immediately and seek medical advice if you experience:

Sudden weakness or numbness

Vision changes or loss

Severe headaches

Confusion or memory problems

Dizziness or balance problems

Speech difficulties

Another stroke or TIA (mini-stroke)

When You Might Not Return to Driving

Some people may not be able to return to driving if they have:

Severe ongoing physical disabilities

Significant visual field defects that cannot be compensated for

Serious cognitive impairment affecting safety

Frequent seizures or other medical complications

Inability to meet the required medical standards even with adaptations

Support and Resources

Useful Contacts:

DVLA Medical Enquiries: 0300 790 6806

Stroke Association: 0303 3033 100

Different Strokes (for younger stroke survivors): 0345 130 7172

Headway (brain injury support): 0808 800 2244

Driving Assessment Centres: Your occupational therapist can refer you to a local driving assessment centre. These centres can:

Assess your fitness to drive

Recommend vehicle adaptations

Provide specialist driving lessons

Issue certificates for DVLA and insurance

Financial Support

You may be eligible for:

Motability Scheme: If you receive certain disability benefits

Access to Work Scheme: Help with travel to work costs

Local authority grants: For vehicle adaptations

Charitable grants: Various charities offer support for mobility needs

Important Reminders

Never drive until you have medical clearance AND DVLA approval

Always inform both DVLA and your insurance company

Regular medical reviews may be required

Consider your own safety and that of other road users

It's better to wait longer than to rush back too early

Taking Alternative Transport

While you cannot drive, consider:

Public transport (you may be eligible for concessionary travel)

Community transport schemes

Hospital transport for medical appointments

Family and friends

Taxi services (some areas offer subsidised schemes)

Cycling (if appropriate and cleared by your medical team)

Remember: This leaflet provides general guidance only. Always follow the specific advice given by your healthcare team and DVLA. Every stroke is different, and your recovery will be individual to you.

For urgent medical concerns, contact your GP or call 999.

This information leaflet is for educational purposes and should not replace professional medical advice.

Gemini 2.5 Flash - Raw Output

(Note: Gemini's output included its own citation numbers in square brackets, which have been changed to full-width brackets 〔〕 to avoid confusion with this article's reference list. All content otherwise unchanged.)

Driving After a Stroke: A Guide for Adults in the UK

Introduction

Having a stroke can be a life-changing event, and one of the questions many people ask is "When can I drive again?" Driving is a complex task that requires good physical, visual, and cognitive abilities, all of which can be affected by a stroke〔1〕〔2〕. This leaflet provides essential information for adults in the UK on the legal requirements and practical considerations for returning to driving safely after a stroke.

Stopping Driving Immediately

By law, you must stop driving immediately after a stroke or Transient Ischaemic Attack (TIA) (often called a 'mini-stroke')〔2〕〔3〕〔4〕〔5〕〔6〕〔7〕〔8〕. This applies regardless of how well you feel or if your symptoms have resolved quickly〔6〕.

The Initial Waiting Period

For most car and motorcycle drivers (Group 1 licence holders), you must not drive for at least one month following a stroke or TIA〔2〕〔3〕〔4〕〔5〕〔8〕〔9〕〔10〕〔11〕〔12〕〔13〕.

You may be able to resume driving after this month if your clinical recovery is satisfactory and you have no residual neurological deficits〔9〕〔10〕〔11〕〔13〕〔14〕.

If you have had multiple TIAs over a short period, you will typically need to wait three months from the last attack before resuming driving〔7〕〔9〕〔10〕〔13〕〔15〕.

If you hold a Large Goods Vehicle (LGV) or Passenger Carrying Vehicle (PCV) licence (Group 2), you must stop driving for at least one year and notify the DVLA immediately〔3〕〔4〕〔5〕〔6〕〔9〕〔10〕〔12〕〔13〕.

Informing the DVLA (Driver and Vehicle Licensing Agency)

It is your legal responsibility to ensure you are fit to drive and to inform the DVLA about any medical condition that could affect your driving〔1〕〔8〕〔9〕〔12〕〔16〕.

For Car and Motorcycle Drivers (Group 1):

You do not need to tell the DVLA if you had a single TIA and have fully recovered, or if you had a stroke and have made a full recovery with no residual problems after one month〔3〕〔5〕〔9〕〔10〕〔11〕〔14〕.

However, you MUST tell the DVLA if any of the following apply one month after your stroke:

You still have weakness in your arms or legs that affects controlling a vehicle〔2〕〔3〕〔5〕〔8〕〔9〕〔17〕.

You have problems with your eyesight, such as visual field loss, double vision, or visual inattention/neglect〔2〕〔3〕〔5〕〔8〕〔9〕〔10〕〔17〕〔18〕.

You have problems with balance, memory, understanding, concentration, or reaction speeds〔1〕〔2〕〔3〕〔5〕〔8〕〔9〕〔17〕〔18〕.

You have had any type of seizure (other than within 24 hours after the stroke, which may have different rules)〔2〕〔3〕〔5〕〔6〕〔7〕〔8〕〔9〕〔11〕〔16〕〔17〕.

You needed brain surgery as part of your stroke treatment (a thrombectomy is generally not counted as brain surgery for this purpose)〔3〕〔5〕〔7〕〔8〕〔9〕〔16〕〔17〕.

You have had more than one stroke or TIA within the past three months〔7〕〔8〕〔9〕〔16〕.

Your doctors have expressed concern about your ability to drive safely〔3〕〔5〕〔7〕〔8〕〔9〕〔16〕.

You can notify the DVLA online at GOV.UK/stroke-and-driving or by phone〔1〕〔6〕〔8〕〔9〕〔14〕〔16〕.

For Bus, Coach, or Lorry Drivers (Group 2):

You MUST tell the DVLA immediately and stop driving for at least one year〔3〕〔4〕〔5〕〔6〕〔9〕〔10〕〔12〕〔13〕〔16〕. You will need to fill in form STR1V〔3〕〔5〕〔14〕〔16〕. Relicensing after one year may be considered with a satisfactory medical report and tests〔6〕〔12〕〔13〕.

Why it's important to inform the DVLA:

Failure to do so can result in a fine of up to £1,000〔3〕〔5〕〔8〕〔9〕〔12〕〔16〕.

You could be prosecuted if you are involved in an accident〔3〕〔5〕〔8〕〔9〕〔12〕〔16〕.

Your vehicle insurance could be invalidated〔2〕〔4〕〔8〕〔9〕〔12〕〔16〕.

Informing Your Insurance Company

You must inform your motor insurance company that you have had a stroke or TIA, regardless of whether you need to tell the DVLA〔1〕〔2〕〔4〕〔6〕〔8〕〔12〕〔16〕〔17〕. Failure to do so could invalidate your insurance〔2〕〔4〕〔12〕〔16〕.

Assessing Your Fitness to Drive

A stroke can affect various skills needed for safe driving, including physical control, vision, attention, concentration, memory, and judgment〔1〕〔2〕.

Medical Review: Discuss your ability to drive with your GP or stroke team. They can provide individual advice and help determine if a formal assessment is needed〔1〕〔2〕〔4〕〔17〕.

Driving Assessments: A Driving Mobility assessment can help confirm your ability to drive safely after a stroke〔1〕〔4〕〔8〕〔9〕〔12〕〔17〕〔18〕〔19〕〔20〕. These centres offer comprehensive assessments which typically include:

A discussion about your medical and driving history〔20〕.

Physical and cognitive assessments (e.g., memory, attention, reaction times)〔1〕〔20〕.

Visual tests specific to driving〔20〕.

An on-road assessment in a dual-controlled car〔20〕.

Your doctor can refer you, or you can self-refer to a Driving Mobility centre〔4〕〔9〕〔17〕.

Vehicle Adaptations

If you have ongoing physical effects from your stroke, vehicle adaptations might help you to drive safely. Modern cars often have features like power steering or automatic transmission that can make driving easier〔9〕〔19〕. Driving Mobility centres can advise on suitable adaptations and approved installers〔9〕〔19〕〔20〕.

Returning to Driving and Building Confidence

Even if medically cleared to drive, many people feel anxious or lack confidence when getting back behind the wheel〔19〕.

Gradual Return: Start with short journeys and gradually increase distance and complexity〔19〕.

Refresher Lessons: Consider taking a few lessons with a qualified driving instructor, especially one who specialises in disability driving〔19〕. They can help you regain confidence and adapt to any changes in your driving.

Occupational Therapy: An occupational therapist can help you prepare for driving again by tailoring your therapy to improve skills needed for driving〔19〕.

Fatigue: Be mindful of fatigue, as it can affect concentration and reaction times〔1〕〔18〕. Plan breaks and drive when you feel most energetic〔19〕.

Further Information and Support

DVLA (Driver and Vehicle Licensing Agency): For official guidance on medical conditions and driving. Visit GOV.UK and search for "Driving after stroke".

Driving Mobility: A network of centres across the UK that offer driving assessments and advice on vehicle adaptations. Visit drivingmobility.org.uk or call 0800 559 3636〔9〕〔12〕〔14〕〔16〕〔19〕〔20〕.

The Stroke Association: Provides information and support for stroke survivors, including advice on driving. Visit stroke.org.uk/driving or call their helpline on 0303 3033 100〔2〕〔8〕〔12〕〔17〕.

Remember, the final decision on whether you can drive rests with the DVLA, based on medical advice and your ability to meet the required standards〔8〕〔16〕. Your safety, and the safety of other road users, is paramount.

Sourceshelp

hacw.nhs.uk

stroke.org.uk

www.gov.uk

uhcw.nhs.uk

www.gov.uk

royaldevon.nhs.uk

motordefencelawyers.co.uk

ncic.nhs.uk

disabilitydrivinginstructors.com

ssahpf.org.uk

www.gov.uk

wyh-allthingsstroke.nhs.uk

ssahpf.org.uk

gmnisdn.org.uk

patient.info

stroke.org.uk

stroke.org.uk

uhd.nhs.uk

stroke.org.uk

drivingmobility.org.uk

DeepSeek v3.1 - Raw Output

Patient Information Leaflet

Driving After a Stroke

Information for drivers in England, Scotland, and Wales

This leaflet explains the rules and steps you need to follow if you want to drive after having a stroke. Your safety and the safety of others is the most important thing.

The Most Important Rule: You Must Tell the DVLA

Having a stroke or a transient ischaemic attack (TIA or 'mini-stroke') will affect your ability to drive. By law, you must inform the DVLA (Driver and Vehicle Licensing Agency).

You must not drive for at least 1 month after a stroke.

You must not drive for at least 1 month after a TIA.

After this one-month period, you can only start driving again if your doctor says it is safe and the DVLA confirms you can.

How to tell the DVLA:

Go to the GOV.UK website and search for "Reporting a medical condition to DVLA".

You will need to fill in a form called a QUESTIONNAIRE FED1-APP1. You can do this online or your GP or hospital team may help you with it.

The DVLA will then contact your doctor and may ask you to have a medical assessment or a driving test.

It is illegal to drive without informing the DVLA. Your insurance will not be valid if you do not tell them.

What Happens Next?

The DVLA will make a decision based on medical advice. This depends on how your stroke has affected you.

If your stroke caused: No lasting physical or cognitive (thinking) problems after 1 month. - What the DVLA will usually do: They may let you drive again without needing a special assessment. They will still need confirmation from your doctor.

If your stroke caused: Problems with limb weakness, vision, sensation, or thinking skills (like concentration, memory, or judgement). - What the DVLA will usually do: They will need more information. They may issue a short-term (1, 2, or 3-year) licence. You will need to renew it regularly so the DVLA can check your health is still safe for driving.

If your stroke caused: More significant disabilities that affect your ability to use a standard car. - What the DVLA will usually do: They may issue a licence on the condition that you only drive a car with specific adaptations (e.g. automatic transmission, steering wheel ball, hand controls).

Getting Back Behind the Wheel

Even if you feel ready and have permission from the DVLA, driving again can be daunting. Here are some steps to help you feel confident and safe.

Talk to Your Healthcare Team: Your doctor, physiotherapist, or occupational therapist can advise you on your readiness. They may refer you for an Occupational Therapy Driving Assessment.

Consider a Professional Assessment: An off-road and on-road assessment with a specialist driving assessor is the best way to know if you are ready. They can check your skills and recommend any car adaptations you might need. You may have to pay for this service.

Start Slowly:

Practice in a quiet, familiar area like an empty car park.

Go for short drives at quiet times of the day.

Have a confident and experienced driver with you for support.

Avoid driving in bad weather, at night, or on busy motorways until you feel completely comfortable.

What About My Car Insurance?

You must also tell your car insurance company about your stroke.

If the DVLA says you can drive, your insurer cannot refuse your insurance or charge you more just because you have had a stroke.

However, if you do not tell them, your policy could be invalid. This means you would not be covered in an accident.

What If I Can't Drive Again?

It can be very difficult to accept if you are told you cannot drive. It’s a big change, but support is available.

Talk to someone: Your GP can provide support and refer you to counselling if you are finding it hard to cope.

Explore other options: You may be eligible for a bus pass, reduced train fares, or community transport schemes.

Contact charities: Organisations like the Stroke Association offer fantastic advice and support for adjusting to life after a stroke.

Key Points to Remember

✅ You MUST tell the DVLA and your insurance company.

✅ You MUST NOT drive for at least 1 month after a stroke or TIA.

✅ Wait for official confirmation from the DVLA before you drive again.

✅ Seek advice from your doctor and consider a professional driving assessment.

✅ Be patient with yourself. It takes time to regain confidence.

Useful Contacts

DVLA Medical Enquiries: 0300 790 6806 / www.gov.uk/contact-dvla

Stroke Association Helpline: 0303 3033 100 / www.stroke.org.uk

Different Strokes (for younger stroke survivors): 0345 130 7172 / www.differentstrokes.co.uk

This leaflet is for information purposes only and does not replace professional medical or legal advice. Rules and regulations can change. Always check the official GOV.UK website for the most up-to-date information.

Microsoft Copilot - Raw Output

🚗 Driving After a Stroke

Patient Information Leaflet (UK)

Why this leaflet matters

Having a stroke or transient ischaemic attack (TIA/mini-stroke) can affect your ability to drive safely. This leaflet explains the rules, your responsibilities, and where to get help.

🛑 Can I drive after a stroke?

Car and motorbike drivers: You must not drive for at least 1 month after a stroke or TIA.

Bus, coach, or lorry drivers: You must not drive for at least 1 year.

After this period, you may only return to driving if you are medically fit and safe to do so.

📋 Your responsibilities

It is your responsibility to make sure you are safe to drive.

You must inform the DVLA (Driver and Vehicle Licensing Agency) if your stroke has left you with ongoing problems such as:

Weakness or poor coordination in your arms or legs

Vision problems (blurred, double vision, or loss of part of your visual field)

Difficulties with memory, concentration, or decision-making

Seizures or brain surgery related to your stroke

You must also tell your insurance company that you have had a stroke or TIA.

👩‍⚕️ Who decides if I can drive again?

Your GP, stroke consultant, or specialist will advise you.

If there are concerns, the DVLA may ask you to attend a driving assessment at a specialist centre.

Occupational Therapists can help assess your physical and cognitive abilities and may refer you for an in-car driving assessment.

✅ Questions to ask yourself before driving again

Have I noticed changes in my vision since my stroke?

Do I feel weaker, slower, or less coordinated?

Do I struggle with concentration, memory, or decision-making?

Do I feel more tired than before?

Has anyone close to me expressed concern about my driving?

If you answer “yes” to any of these, speak to your GP before driving.

📞 Useful contacts

DVLA Medical Enquiries: 0300 790 6806

Stroke Association Helpline: 0303 3033 100 | stroke.org.uk/driving

Driving Mobility Centres: drivingmobility.org.uk - for driving assessments and advice

⚠️ Key points to remember

Do not drive for at least 1 month after a stroke or TIA (1 year for bus/lorry drivers).

Always check with your doctor before returning to driving.

Inform the DVLA and your insurance company if you have ongoing problems.

Safety comes first - for you, your passengers, and others on the road.

ChatGPT-5o (Standard) - Raw Output

Driving After Stroke

Information for Patients and Carers

Version: 1.0 Date: [Insert date] Review due: [Insert date]

What is the purpose of this leaflet?

This leaflet aims to:

Explain when you may safely consider returning to driving after a stroke

Highlight legal and medical responsibilities you have

Describe how stroke-related changes can affect driving

Offer guidance on assessments, supports and next steps

It is not a substitute for personalised medical advice. Always check with your stroke team, GP, occupational therapist or the Driver and Vehicle Licensing Agency (DVLA) in your case.

Key Rules & Legal Requirements in the UK

Stop driving immediately after a stroke or transient ischaemic attack (TIA). GOV.UK+2stroke.org.uk+2

You must not drive for at least one calendar month (28 days) after the event. UHCW+3UHD+3GOV.UK+3

You do not need to inform DVLA during that first month (unless your hospital/clinician does so under duty of care). UHCW+2stroke.org.uk+2

After one month, if you have any residual effects from your stroke that may influence your fitness to drive, you must notify DVLA using the medical form STR1 (for car/motorcycle licences). GOV.UK+2stroke.org.uk+2

If your licence is for bus, coach or lorry (Group 2 licence), you must notify DVLA immediately and you are disqualified from driving for at least one year. GOV.UK+2ssahpf.org.uk+2

Failing to notify the DVLA when required may lead to a fine (up to £1,000) or prosecution, especially if you are involved in a road traffic accident. GOV.UK+2stroke.org.uk+2

Inform your motor insurance company about your stroke or TIA. Driving against medical advice or without disclosure may invalidate your insurance. ssahpf.org.uk+3UHCW+3stroke.org.uk+3

How Stroke Can Affect Driving Ability

A stroke can affect one or more of the following, all of which are relevant for driving safely:

Function: Motor & Sensory (strength, coordination, sensation). Possible Effects After Stroke: Weakness or numbness in arm(s) or leg(s); reduced coordination; slower reaction times. Why It Matters for Driving: Difficulty controlling the steering wheel, pedals, clutch, brakes or shifting gears.

Function: Vision & Visual Processing. Possible Effects After Stroke: Blind spots or visual field loss, double vision, difficulty with eye movements, visual inattention/neglect. Why It Matters for Driving: Missing hazards from one side; delayed response to movement; misjudging distances UHCW+3British and Irish Orthoptic Society+3ssahpf.org.uk+3.

Function: Cognitive / Executive Functions. Possible Effects After Stroke: Reduced attention, slower thinking, problems planning, memory or decision-making, impaired concentration. Why It Matters for Driving: Delayed reaction to changing traffic; trouble multitasking; forgetting road rules.

Function: Fatigue & Endurance. Possible Effects After Stroke: Extreme tiredness, fluctuating energy levels, mental fatigue. Why It Matters for Driving: Driving demands sustained focus over time; fatigue reduces vigilance.

Function: Communication / Language. Possible Effects After Stroke: Difficulty understanding written / verbal instructions, aphasia, poor processing of traffic signs. Why It Matters for Driving: Trouble interpreting road signs or instructions from other road users.

Function: Other Effects. Possible Effects After Stroke: Balance problems, dizziness, seizures (if they occur after stroke), medication side-effects. Why It Matters for Driving: These may pose safety risks under driving conditions.

Because recovery may continue for many weeks or months, some impairments might improve over time. However, persistent deficits may influence whether it is safe (or legal) to resume driving.

Steps to Return to Driving: What You Should Do

Wait the one-month minimum period before considering driving.

Discuss return to driving with your GP, stroke specialist or occupational therapist once you feel ready.

Undergo assessments to evaluate whether your physical, cognitive and visual abilities are safe for driving.

Occupational therapists may carry out screening tests. hacw.nhs.uk+2UHD+2

You may be referred for a specialist driving assessment (on-road test, simulator, etc.) at a centre affiliated with Driving Mobility or equivalent. UHD+3ssahpf.org.uk+3stroke.org.uk+3

Vision assessments (by optometrist or orthoptist) should check visual acuity, eye movements, visual fields and binocular vision. British and Irish Orthoptic Society+2ssahpf.org.uk+2

Obtain a medical report / submit information to DVLA using the required forms (e.g. STR1 for Group 1 licences). stroke.org.uk+2ssahpf.org.uk+2

Await DVLA decision - they may accept your licence as is, ask for further tests, impose restrictions, issue a time-limited licence, or refuse or withdraw your licence. GOV.UK+3ncic.nhs.uk+3stroke.org.uk+3

Inform your insurance provider before driving again.

If necessary, consider modified driving or adapted vehicle controls, or explore alternative transport options (public transport, community services). ssahpf.org.uk+1

What “Ongoing Effects” Require You to Tell DVLA?

After one month, you must notify DVLA if you still have any of the following:

Weakness in arms or legs, or loss of coordination that may affect control of vehicle

Visual problems: double vision, field loss, visual neglect, or inability to meet eyesight standards for driving British and Irish Orthoptic Society+3GOV.UK+3stroke.org.uk+3

Problems with memory, understanding or concentration

Dizziness, balance problems or unexplained falls

Seizure occurring after more than 24 hours from stroke onset (or any unprovoked seizure)

Brain surgery as part of stroke treatment

Multiple strokes or TIAs occurring in a short timeframe

If your clinician (GP, consultant) expresses concern about your fitness to drive UHD+3GOV.UK+3ssahpf.org.uk+3

If one or more of these applies, DVLA must be informed, and they will review your case. GOV.UK+2ssahpf.org.uk+2

What Might DVLA Decide?

Based on the medical information and assessments submitted, DVLA may:

Let you continue driving normally

Grant you a time-limited licence (e.g. valid for a few years, subject to reevaluation)

Require you to take further tests (vision test, driving assessment)

Impose driving restrictions (e.g. only automatic transmission, no motorway driving)

Withdraw or refuse your licence

In some cases with stable visual or other impairments, allow you to apply as an “exceptional case” (rare and strictly regulated) UHCW+3ssahpf.org.uk+3stroke.org.uk+3

Safety Tips & Considerations

Do not rush the return to driving - premature return may put yourself and others at risk

Start with short, familiar journeys and avoid busy motorways or peak traffic

Consider a supervised drive with a driving instructor or occupational therapist first

Be extra cautious in poor weather, low light or unfamiliar roads

Monitor and manage fatigue, ensure good rest, avoid drowsy medications

Make sure your vision correction (glasses/contact lenses) is up to date

Always follow DVLA and insurance instructions

If your conditions worsen or new symptoms arise, stop driving and seek advice

When You Cannot Drive (or May Be Permanently Unsuitable)

Even with full rehabilitation, some people may never be safe to drive again. This may be the case if:

Significant and permanent vision defects or visual neglect

Severe cognitive impairment affecting judgement, reaction times or multitasking

Uncontrolled seizures

Persistent motor/sensory impairments that cannot be adapted for

Ongoing dizziness or balance problems

Inability to meet DVLA standards even with adaptations

If you cannot drive, your therapy and care team can help you find alternative transport solutions (community transport, assistance schemes, volunteer services, adapted taxis, etc.).

Useful Contacts & Resources

DVLA Medical Enquiries - Tel: 0300 790 6806 UHCW+3GOV.UK+3stroke.org.uk+3

DVLA medical information (online) - gov.uk/stroke-and-driving GOV.UK+1

Driver and Vehicle Licensing Agency (DVLA), Swansea - address on STR1 form stroke.org.uk+2UHCW+2

Driving assessment centres / Driving Mobility - many regions have specialist driving assessment services ssahpf.org.uk+2stroke.org.uk+2

Stroke Association (UK) - for general support and information stroke.org.uk+1

Regional NHS stroke / rehabilitation services - your local hospital or community team

Frequently Asked Questions (FAQs)

Question: I feel completely "back to normal" before one month is over. Can I drive? Answer: No. The law mandates a minimum no-drive period of one month, regardless of how well you feel. GOV.UK+2stroke.org.uk+2

Question: Do I always have to send in my driving licence to DVLA? Answer: Not necessarily. Usually you only need to provide a medical report and relevant forms (like STR1); DVLA may issue decisions without you surrendering your licence. stroke.org.uk+2ssahpf.org.uk+2

Question: If I had a TIA (mini-stroke), do the same rules apply? Answer: Yes. You still must not drive for one month after a TIA. However, if you recover fully, you may not need to notify DVLA. GOV.UK+2UHCW+2

Question: What if I had multiple TIAs in a short period? Answer: In that case, a 3-month no-drive period may be required. stroke.org.uk+3ssahpf.org.uk+3eastmidlandsisdn.nhs.uk+3

Question: If I have a Group 2 (bus/lorry) licence, are the rules different? Answer: Yes - you must inform DVLA immediately, and you are disqualified from driving for at least one year. stroke.org.uk+3GOV.UK+3ssahpf.org.uk+3

Summary (What You Must Remember)

Stop driving straight away after a stroke or TIA.

Do not drive for at least one month.

After one month, if you have any continuing issues that affect safe driving, notify DVLA.

Undergo assessments (physical, vision, cognitive) before returning.

DVLA will review and decide whether (and how) you may drive.

Always inform your insurance company.

If driving is no longer safe or allowed, explore alternative transport options.

ChatGPT-5 (Thinking) - Raw Output

Driving After a Stroke (UK)

Patient information for adults with a car or motorcycle licence (Group 1) and bus/lorry licence (Group 2). Last reviewed: 27 September 2025

The short answer

Do not drive for at least 1 month after a stroke or TIA (mini-stroke). GOV.UK+1

You may drive again after 1 month only if you’ve made a satisfactory recovery and no problems remain that could affect safe driving (such as visual field loss, significant weakness, neglect, seizures or serious cognitive problems). If problems remain at 1 month, tell the DVLA. stroke.org.uk+1

Who must tell the DVLA?

Group 1 (car/motorcycle)

Stroke: You must stop driving for 1 month. If any deficits remain after a month (e.g., visual field loss, limb weakness, neglect, seizures, or cognitive problems), you must notify DVLA and should not drive until DVLA says you can. GOV.UK+1

TIA: Do not drive for 1 month. If you’ve fully recovered and have no further TIAs, you usually don’t need to notify DVLA; do tell your insurer. If symptoms persist beyond a month, notify DVLA. GOV.UK+1

Group 2 (bus/lorry)

Stroke or TIA: You must tell DVLA and stop driving. Typical minimum off-driving period is at least 1 year, with DVLA assessment before any return. Use form STR1V to notify. GOV.UK

Specific issues that can delay return to driving

Seizure soon after stroke (a “provoked” seizure): You must stop driving and notify DVLA. Usual minimum off-driving period is 6 months for Group 1 (longer for Group 2). GOV.UK

Visual field loss (e.g., hemianopia): Often not compatible with Group 1 standards; DVLA usually requires a binocular Esterman field test. Some “exceptional case” relicensing may be possible in strictly defined circumstances. Do not drive and notify DVLA. GOV.UK+1

Cognitive problems (memory, attention, processing): Notify DVLA; you must be safe to drive. GOV.UK

Getting back on the road-step by step (Group 1)

First month: Do not drive. Focus on recovery and rehabilitation. GOV.UK

At ~4 weeks: Speak to your stroke team/GP about fitness to drive. If no deficits affect driving, you can usually resume. If deficits remain, notify DVLA and do not drive. GMNISDN

Insurance: Inform your insurer that you’ve had a stroke/TIA-driving without declaring relevant medical conditions may invalidate cover. (Insurers follow DVLA rules.) stroke.org.uk

If in doubt: Ask about a Driving Mobility assessment (specialist on-road/rig assessment and adaptation advice). mobility-centres.org.uk

Practical tips

Vision: Book an optician/orthoptic review if you notice new blurring, double vision or field loss. British and Irish Orthoptic Society

Fatigue & attention: Only return when you can concentrate for sustained periods without excessive fatigue. Many hospitals provide checklists; ask your stroke team. UHD

Adaptations: If you have residual physical limitations, vehicle adaptations and specialist driving tuition may help; the DVLA can consider licences with conditions. mobility-centres.org.uk

How to notify DVLA

Online guidance: Search “GOV.UK stroke and driving” for the current pages and online forms. GOV.UK

Group 2 (bus/lorry): Use form STR1V to report a stroke/TIA. GOV.UK

When should I seek urgent advice?

New symptoms (weakness, speech/vision problems), any seizure, or another TIA: stop driving and contact your stroke team/111/999 as appropriate. For multiple TIAs over a short period, longer off-driving periods may apply-seek medical advice. disabilitydrivinginstructors.com

Key standards and where this guidance comes from

DVLA: Stroke/TIA & medical standards (latest “Assessing fitness to drive” guide-August 2024 update). GOV.UK+1

GOV.UK pages for stroke/TIA and driving. GOV.UK+2GOV.UK+2

Stroke Association-patient-friendly guidance. stroke.org.uk+1

Visual standards & Esterman testing (DVLA). GOV.UK

Disclaimer

This leaflet summarises UK rules as of 27 Sep 2025. DVLA standards can change and individual cases vary. Always follow advice from your stroke team/GP and the DVLA’s latest guidance. If you’re unsure, don’t drive until you’ve checked.

Appendix B

Standardised Text Used for Analysis

The texts below are the standardised versions of the seven LLM-generated leaflets used for analysis. The Stroke Association leaflet (reference [1]) is not reproduced here due to copyright but was standardised using the identical protocol (Appendix C) for comparative evaluation. The original source is publicly accessible via the reference list. Readability scores reported in Results were computed from these texts on 27 September 2025 using readabilityformulas.com. Minor re-analysis variation may occur across tools/time due to parsing differences.

Perplexity AI (free) - Standardised Text

Driving After Stroke: Patient Information Leaflet for Adults in the UK

Important Safety Information: Do not drive for at least 1 month following a stroke or transient ischemic attack (TIA or mini-stroke). For commercial drivers (e.g., lorry, bus), the minimum driving ban is 1 year. You do not need to inform the Driver and Vehicle Licensing Agency (DVLA) within the first month, but after one month, you must inform them if you have ongoing effects from your stroke that could affect driving.

Effects That Must Be Reported to DVLA After One Month: Notify the DVLA if you have any of the following ongoing symptoms affecting your driving ability: Weakness or lack of coordination in arms or legs. Vision problems such as visual field loss, double vision, or visual neglect. Problems with memory, understanding, concentration, or slower reaction times. Ongoing tiredness or cognitive problems. Seizures after the stroke (except for seizures within 24 hours of the stroke). Brain surgery related to stroke treatment. Multiple strokes within the past three months. Any professional medical concerns about your driving ability. If you hold a Group 2 licence (professional drivers).

Telling Your Insurance Company: It is essential to inform your insurance company that you have had a stroke or TIA. Failure to do so may make your insurance invalid.

Returning to Driving: After the initial 1-month period, discuss with your GP or healthcare professional whether you are fit to resume driving. You may be referred for a driving assessment to evaluate your fitness to drive safely. Driving assessments can also be self-referred and help determine if vehicle adaptations or further rehabilitation are needed.

Vision After Stroke: Visit an optician to check your vision if you have concerns post-stroke. Some visual impairments may require formal assessments before returning to driving.

Additional Notes: If you have had multiple TIAs, you must not drive for 1 month after each episode. For seizures related to stroke, specific rules apply, including driving restrictions for 6 to 12 months depending on the seizure type. If you experience cognitive or language difficulties such as aphasia, speak to your healthcare provider for guidance on driving safety.

Support and Resources: Driving Mobility and local driving assessment centres offer specialised assessments and advice. Speak to stroke support services for additional help navigating your return to driving.

This leaflet aims to support safe and responsible driving after a stroke while complying with UK legal and medical regulations. Always consult your healthcare provider for personalised advice. If needed, a printable version of official guidance is available from NHS and stroke charity websites.

Claude Sonnet 4 - Standardised Text

Driving After Stroke:

Patient Information Leaflet:

Understanding Your Situation: Having a stroke can be life-changing, and you may be wondering when or if you'll be able to drive again. This leaflet provides essential information about returning to driving safely and legally after a stroke. The good news: Many people do return to driving after stroke, but it's important to follow the proper steps to ensure your safety and that of other road users.

Immediate Action Required: You must stop driving immediately after a stroke. This is both a legal requirement and essential for safety. You cannot drive for at least one month after a stroke, and in many cases longer.

Legal Requirements in the UK:

DVLA Notification: You must inform the Driver and Vehicle Licensing Agency (DVLA) about your stroke. This must be done as soon as possible after your stroke. Failure to inform DVLA is a criminal offence. Contact DVLA on 0300 790 6806 or visit gov.uk/stroke-and-driving.

Minimum Waiting Periods: Car or motorcycle licence: Cannot drive for at least 1 month after stroke. Bus, coach, or lorry licence: Cannot drive for at least 12 months after stroke. These are minimum periods; you may need to wait longer depending on your recovery.

Medical Assessment: Before you can return to driving, you need:

Healthcare Team Clearance: Your doctor, occupational therapist, or stroke specialist must assess your fitness to drive. They will evaluate your physical abilities, vision, cognitive function, and reaction times. You may need multiple assessments as you recover.

Possible Assessments Include: Physical examination of strength, coordination, and mobility. Vision tests (including visual field testing). Cognitive assessments (memory, concentration, decision-making). Reaction time testing. On-road driving assessment with a specialist instructor.

Common Stroke Effects That May Affect Driving:

Physical Effects: Weakness or paralysis on one side of the body. Reduced coordination and balance. Fatigue and reduced stamina.

Vision Problems: Loss of visual field (hemianopia). Double vision. Difficulty judging distances.

Cognitive Effects: Problems with concentration and attention. Memory difficulties. Slower processing of information. Difficulty making quick decisions.

Communication: Speech problems do not necessarily prevent driving, but severe difficulties may affect safety.

Vehicle Adaptations:

Common Adaptations: Hand controls for acceleration and braking. Steering wheel knobs or handles. Left foot accelerator pedals. Mirrors to compensate for visual field loss. Seat modifications for better positioning.

Getting Adaptations: Speak to an occupational therapist or driving assessment specialist. Some adaptations may be funded through local authority grants or charities. All adaptations must be approved and properly fitted.

Insurance Considerations:

Informing Your Insurer: You must tell your car insurance company about your stroke. This should be done immediately, even before you return to driving. Your premium may change, but driving without disclosure could invalidate your policy. Shop around as some insurers specialise in covering drivers with medical conditions.

The Return to Driving Process: Step 1: Receive medical clearance from your healthcare team. Step 2: Get approval from DVLA (they may request medical reports). Step 3: Inform your insurance company. Step 4: Consider a driving assessment with a specialist instructor. Step 5: Start with short, familiar journeys in good conditions. Step 6: Gradually build up your driving as confidence and ability improve.

Warning Signs to Stop Driving: Sudden weakness or numbness. Vision changes or loss. Severe headaches. Confusion or memory problems. Dizziness or balance problems. Speech difficulties. Another stroke or TIA (mini-stroke).

When You Might Not Return to Driving: Severe ongoing physical disabilities. Significant visual field defects that cannot be compensated for. Serious cognitive impairment affecting safety. Frequent seizures or other medical complications. Inability to meet the required medical standards even with adaptations.

Support and Resources:

Useful Contacts: DVLA Medical Enquiries: 0300 790 6806. Stroke Association: 0303 3033 100. Different Strokes (for younger stroke survivors): 0345 130 7172. Headway (brain injury support): 0808 800 2244.

Driving Assessment Centres: Your occupational therapist can refer you to a local driving assessment centre. These centres can assess your fitness to drive, recommend vehicle adaptations, provide specialist driving lessons, and issue certificates for DVLA and insurance.

Financial Support: You may be eligible for the Motability Scheme if you receive certain disability benefits. Access to Work Scheme may help with travel to work costs. Local authority grants may support vehicle adaptations. Charitable grants are available from various organisations for mobility needs.

Important Reminders: Never drive until you have medical clearance and DVLA approval. Always inform both DVLA and your insurance company. Regular medical reviews may be required. Consider your own safety and that of other road users. It is better to wait longer than to rush back too early.

Taking Alternative Transport: Public transport (you may be eligible for concessionary travel). Community transport schemes. Hospital transport for medical appointments. Family and friends. Taxi services (some areas offer subsidised schemes). Cycling (if appropriate and cleared by your medical team).

Remember: This leaflet provides general guidance only. Always follow the specific advice given by your healthcare team and DVLA. Every stroke is different, and your recovery will be individual to you. For urgent medical concerns, contact your GP or call 999. This information leaflet is for educational purposes and should not replace professional medical advice.

Gemini 2.5 Flash - Standardised Text

Driving After a Stroke: A Guide for Adults in the UK:

Introduction: Having a stroke can be a life-changing event, and one of the questions many people ask is "When can I drive again?". Driving is a complex task that requires good physical, visual, and cognitive abilities, all of which can be affected by a stroke. This leaflet provides essential information for adults in the UK on the legal requirements and practical considerations for returning to driving safely after a stroke.

Stopping Driving Immediately: By law, you must stop driving immediately after a stroke or Transient Ischaemic Attack (TIA) (often called a 'mini-stroke'). This applies regardless of how well you feel or if your symptoms have resolved quickly.

The Initial Waiting Period: For most car and motorcycle drivers (Group 1 licence holders), you must not drive for at least one month following a stroke or TIA. You may be able to resume driving after this month if your clinical recovery is satisfactory and you have no residual neurological deficits. If you have had multiple TIAs over a short period, you will typically need to wait three months from the last attack before resuming driving. If you hold a Large Goods Vehicle (LGV) or Passenger Carrying Vehicle (PCV) licence (Group 2), you must stop driving for at least one year and notify the DVLA immediately.

Informing the DVLA (Driver and Vehicle Licensing Agency): It is your legal responsibility to ensure you are fit to drive and to inform the DVLA about any medical condition that could affect your driving.

For Car and Motorcycle Drivers (Group 1): You do not need to tell the DVLA if you had a single TIA and have fully recovered, or if you had a stroke and have made a full recovery with no residual problems after one month. However, you MUST tell the DVLA if any of the following apply one month after your stroke: You still have weakness in your arms or legs that affects controlling a vehicle. You have problems with your eyesight, such as visual field loss, double vision, or visual inattention/neglect. You have problems with balance, memory, understanding, concentration, or reaction speeds. You have had any type of seizure (other than within 24 hours after the stroke, which may have different rules). You needed brain surgery as part of your stroke treatment (a thrombectomy is generally not counted as brain surgery for this purpose). You have had more than one stroke or TIA within the past three months. Your doctors have expressed concern about your ability to drive safely. You can notify the DVLA online at GOV.UK/stroke-and-driving or by phone.

For Bus, Coach, or Lorry Drivers (Group 2): You MUST tell the DVLA immediately and stop driving for at least one year. You will need to fill in form STR1V. Relicensing after one year may be considered with a satisfactory medical report and tests.

Why it's important to inform the DVLA: Failure to do so can result in a fine of up to £1,000. You could be prosecuted if you are involved in an accident. Your vehicle insurance could be invalidated.

Informing Your Insurance Company: You must inform your motor insurance company that you have had a stroke or TIA, regardless of whether you need to tell the DVLA. Failure to do so could invalidate your insurance.

Assessing Your Fitness to Drive: A stroke can affect various skills needed for safe driving, including physical control, vision, attention, concentration, memory, and judgment.

Medical Review: Discuss your ability to drive with your GP or stroke team. They can provide individual advice and help determine if a formal assessment is needed.

Driving Assessments: A Driving Mobility assessment can help confirm your ability to drive safely after a stroke. These centres offer comprehensive assessments which typically include: A discussion about your medical and driving history. Physical and cognitive assessments (e.g., memory, attention, reaction times). Visual tests specific to driving. An on-road assessment in a dual-controlled car. Your doctor can refer you, or you can self-refer to a Driving Mobility centre.

Vehicle Adaptations: If you have ongoing physical effects from your stroke, vehicle adaptations might help you to drive safely. Modern cars often have features like power steering or automatic transmission that can make driving easier. Driving Mobility centres can advise on suitable adaptations and approved installers.

Returning to Driving and Building Confidence: Even if medically cleared to drive, many people feel anxious or lack confidence when getting back behind the wheel.

Gradual Return: Start with short journeys and gradually increase distance and complexity.

Refresher Lessons: Consider taking a few lessons with a qualified driving instructor, especially one who specialises in disability driving. They can help you regain confidence and adapt to any changes in your driving.

Occupational Therapy: An occupational therapist can help you prepare for driving again by tailoring your therapy to improve skills needed for driving.

Fatigue: Be mindful of fatigue, as it can affect concentration and reaction times. Plan breaks and drive when you feel most energetic.

Further Information and Support: DVLA (Driver and Vehicle Licensing Agency): For official guidance on medical conditions and driving. Visit GOV.UK and search for "Driving after stroke". Driving Mobility: A network of centres across the UK that offer driving assessments and advice on vehicle adaptations. Visit drivingmobility.org.uk or call 0800 559 3636. The Stroke Association: Provides information and support for stroke survivors, including advice on driving. Visit stroke.org.uk/driving or call their helpline on 0303 3033 100.

Remember: The final decision on whether you can drive rests with the DVLA, based on medical advice and your ability to meet the required standards. Your safety, and the safety of other road users, is paramount.

DeepSeek v3.1 - Standardised Text

Patient Information Leaflet:

Driving After a Stroke:

Information for drivers in England, Scotland, and Wales: This leaflet explains the rules and steps you need to follow if you want to drive after having a stroke. Your safety and the safety of others is the most important thing.

The Most Important Rule: You Must Tell the DVLA Having a stroke or a transient ischaemic attack (TIA or 'mini-stroke') will affect your ability to drive. By law, you must inform the DVLA (Driver and Vehicle Licensing Agency). You must not drive for at least 1 month after a stroke. You must not drive for at least 1 month after a TIA. After this one-month period, you can only start driving again if your doctor says it is safe and the DVLA confirms you can.

How to tell the DVLA: Go to the GOV.UK website and search for "Reporting a medical condition to DVLA". You will need to fill in a form called a QUESTIONNAIRE FED1-APP1. You can do this online or your GP or hospital team may help you with it. The DVLA will then contact your doctor and may ask you to have a medical assessment or a driving test. It is illegal to drive without informing the DVLA. Your insurance will not be valid if you do not tell them.

What Happens Next? The DVLA will make a decision based on medical advice. This depends on how your stroke has affected you. If your stroke caused no lasting physical or cognitive (thinking) problems after 1 month, they may let you drive again without needing a special assessment, and they will still need confirmation from your doctor. If your stroke caused problems with limb weakness, vision, sensation, or thinking skills (like concentration, memory, or judgement), they will need more information, and they may issue a short-term (1, 2, or 3-year) licence that you will need to renew regularly so the DVLA can check your health is still safe for driving. If your stroke caused more significant disabilities that affect your ability to use a standard car, they may issue a licence on the condition that you only drive a car with specific adaptations (e.g. automatic transmission, steering wheel ball, hand controls).

Getting Back Behind the Wheel: Even if you feel ready and have permission from the DVLA, driving again can be daunting. Here are some steps to help you feel confident and safe. Talk to Your Healthcare Team: Your doctor, physiotherapist, or occupational therapist can advise you on your readiness. They may refer you for an Occupational Therapy Driving Assessment. Consider a Professional Assessment: An off-road and on-road assessment with a specialist driving assessor is the best way to know if you are ready. They can check your skills and recommend any car adaptations you might need. You may have to pay for this service. Start Slowly: Practice in a quiet, familiar area like an empty car park. Go for short drives at quiet times of the day. Have a confident and experienced driver with you for support. Avoid driving in bad weather, at night, or on busy motorways until you feel completely comfortable.

What About My Car Insurance? You must also tell your car insurance company about your stroke. If the DVLA says you can drive, your insurer cannot refuse your insurance or charge you more just because you have had a stroke. However, if you do not tell them, your policy could be invalid. This means you would not be covered in an accident.

What If I Can't Drive Again? It can be very difficult to accept if you are told you cannot drive. It’s a big change, but support is available. Talk to someone: Your GP can provide support and refer you to counselling if you are finding it hard to cope. Explore other options: You may be eligible for a bus pass, reduced train fares, or community transport schemes. Contact charities: Organisations like the Stroke Association offer fantastic advice and support for adjusting to life after a stroke.

Key Points to Remember: You MUST tell the DVLA and your insurance company. You MUST NOT drive for at least 1 month after a stroke or TIA. Wait for official confirmation from the DVLA before you drive again. Seek advice from your doctor and consider a professional driving assessment. Be patient with yourself. It takes time to regain confidence.

Useful Contacts: DVLA Medical Enquiries: 0300 790 6806 / www.gov.uk/contact-dvla. Stroke Association Helpline: 0303 3033 100 / www.stroke.org.uk. Different Strokes (for younger stroke survivors): 0345 130 7172 / www.differentstrokes.co.uk.

This leaflet is for information purposes only and does not replace professional medical or legal advice. Rules and regulations can change. Always check the official GOV.UK website for the most up-to-date information.

Microsoft Copilot - Standardised Text

Driving After a Stroke:

Patient Information Leaflet (UK):

Why this leaflet matters: Having a stroke or transient ischaemic attack (TIA/mini-stroke) can affect your ability to drive safely. This leaflet explains the rules, your responsibilities, and where to get help.

Can I drive after a stroke?: Car and motorbike drivers: You must not drive for at least 1 month after a stroke or TIA. Bus, coach, or lorry drivers: You must not drive for at least 1 year. After this period, you may only return to driving if you are medically fit and safe to do so.

Your responsibilities: It is your responsibility to make sure you are safe to drive. You must inform the DVLA (Driver and Vehicle Licensing Agency) if your stroke has left you with ongoing problems such as: Weakness or poor coordination in your arms or legs. Vision problems (blurred, double vision, or loss of part of your visual field). Difficulties with memory, concentration, or decision-making. Seizures or brain surgery related to your stroke. You must also tell your insurance company that you have had a stroke or TIA.

Who decides if I can drive again?: Your GP, stroke consultant, or specialist will advise you. If there are concerns, the DVLA may ask you to attend a driving assessment at a specialist centre. Occupational therapists can help assess your physical and cognitive abilities and may refer you for an in-car driving assessment.

Questions to ask yourself before driving again: Have I noticed changes in my vision since my stroke? Do I feel weaker, slower, or less coordinated? Do I struggle with concentration, memory, or decision-making? Do I feel more tired than before? Has anyone close to me expressed concern about my driving? If you answer “yes” to any of these, speak to your GP before driving.

Useful contacts: DVLA Medical Enquiries: 0300 790 6806. Stroke Association Helpline: 0303 3033 100 | stroke.org.uk/driving. Driving Mobility Centres: drivingmobility.org.uk - for driving assessments and advice.

Key points to remember: Do not drive for at least 1 month after a stroke or TIA (1 year for bus/lorry drivers). Always check with your doctor before returning to driving. Inform the DVLA and your insurance company if you have ongoing problems. Safety comes first - for you, your passengers, and others on the road.

ChatGPT-5o (Standard) - Standardised Text

Driving After Stroke:

Information for Patients and Carers:

Version: 1.0. Date: [Insert date]. Review due: [Insert date].

What is the purpose of this leaflet?: This leaflet aims to: Explain when you may safely consider returning to driving after a stroke. Highlight legal and medical responsibilities you have. Describe how stroke-related changes can affect driving. Offer guidance on assessments, supports and next steps. It is not a substitute for personalised medical advice. Always check with your stroke team, GP, occupational therapist or the Driver and Vehicle Licensing Agency (DVLA) in your case.

Key Rules & Legal Requirements in the UK: Stop driving immediately after a stroke or transient ischaemic attack (TIA). You must not drive for at least one calendar month (28 days) after the event. You do not need to inform DVLA during that first month (unless your hospital or clinician does so under duty of care). After one month, if you have any residual effects from your stroke that may influence your fitness to drive, you must notify DVLA using the medical form STR1 (for car or motorcycle licences). If your licence is for bus, coach or lorry (Group 2 licence), you must notify DVLA immediately and you are disqualified from driving for at least one year. Failing to notify the DVLA when required may lead to a fine of up to £1,000 or prosecution, especially if you are involved in a road traffic accident. Inform your motor insurance company about your stroke or TIA. Driving against medical advice or without disclosure may invalidate your insurance.

How Stroke Can Affect Driving Ability: Function: Motor & Sensory (strength, coordination, sensation). Possible Effects After Stroke: Weakness or numbness in arm(s) or leg(s); reduced coordination; slower reaction times. Why It Matters for Driving: Difficulty controlling the steering wheel, pedals, clutch, brakes or shifting gears. Function: Vision & Visual Processing. Possible Effects After Stroke: Blind spots or visual field loss, double vision, difficulty with eye movements, visual inattention/neglect. Why It Matters for Driving: Missing hazards from one side; delayed response to movement; misjudging distances. Function: Cognitive / Executive Functions. Possible Effects After Stroke: Reduced attention, slower thinking, problems planning, memory or decision-making, impaired concentration. Why It Matters for Driving: Delayed reaction to changing traffic; trouble multitasking; forgetting road rules. Function: Fatigue & Endurance. Possible Effects After Stroke: Extreme tiredness, fluctuating energy levels, mental fatigue. Why It Matters for Driving: Driving demands sustained focus over time; fatigue reduces vigilance. Function: Communication / Language. Possible Effects After Stroke: Difficulty understanding written / verbal instructions, aphasia, poor processing of traffic signs. Why It Matters for Driving: Trouble interpreting road signs or instructions from other road users. Function: Other Effects. Possible Effects After Stroke: Balance problems, dizziness, seizures (if they occur after stroke), medication side-effects. Why It Matters for Driving: These may pose safety risks under driving conditions. Because recovery may continue for many weeks or months, some impairments might improve over time. However, persistent deficits may influence whether it is safe or legal to resume driving.

Steps to Return to Driving: What You Should Do: Wait the one-month minimum period before considering driving. Discuss return to driving with your GP, stroke specialist or occupational therapist once you feel ready. Undergo assessments to evaluate whether your physical, cognitive and visual abilities are safe for driving. Occupational therapists may carry out screening tests. You may be referred for a specialist driving assessment (on-road test, simulator, etc.) at a centre affiliated with Driving Mobility or equivalent. Vision assessments by an optometrist or orthoptist should check visual acuity, eye movements, visual fields and binocular vision. Obtain a medical report and submit information to DVLA using the required forms (for example, STR1 for Group 1 licences). Await DVLA decision - they may accept your licence as is, ask for further tests, impose restrictions, issue a time-limited licence, or refuse or withdraw your licence. Inform your insurance provider before driving again. If necessary, consider modified driving or adapted vehicle controls, or explore alternative transport options such as public transport and community services.

What “Ongoing Effects” Require You to Tell DVLA?: After one month, you must notify DVLA if you still have any of the following. Weakness in arms or legs, or loss of coordination that may affect control of a vehicle. Visual problems such as double vision, field loss, visual neglect, or inability to meet eyesight standards for driving. Problems with memory, understanding or concentration. Dizziness, balance problems or unexplained falls. A seizure occurring after more than 24 hours from stroke onset, or any unprovoked seizure. Brain surgery as part of stroke treatment. Multiple strokes or TIAs occurring in a short timeframe. Concern from your clinician about your fitness to drive. If one or more of these applies, DVLA must be informed, and they will review your case.

What Might DVLA Decide?: Based on the medical information and assessments submitted, DVLA may: Let you continue driving normally. Grant you a time-limited licence subject to re-evaluation. Require you to take further tests such as a vision test or driving assessment. Impose driving restrictions such as only automatic transmission. Withdraw or refuse your licence. In some cases with stable visual or other impairments, allow application as an exceptional case, which is rare and strictly regulated.

Safety Tips & Considerations: Do not rush the return to driving; premature return may put you and others at risk. Start with short, familiar journeys and avoid busy motorways or peak traffic. Consider a supervised drive with a driving instructor or occupational therapist first. Be cautious in poor weather, low light or unfamiliar roads. Monitor and manage fatigue, ensure good rest, and avoid drowsy medications. Make sure your vision correction is up to date. Always follow DVLA and insurance instructions. If your condition worsens or new symptoms arise, stop driving and seek advice.

When You Cannot Drive (or May Be Permanently Unsuitable): Even with rehabilitation, some people may never be safe to drive again. This may be the case if you have: Significant and permanent vision defects or visual neglect. Severe cognitive impairment affecting judgement, reaction times or multitasking. Uncontrolled seizures. Persistent motor or sensory impairments that cannot be adapted for. Ongoing dizziness or balance problems. Inability to meet DVLA standards even with adaptations. If you cannot drive, your therapy and care team can help you find alternative transport solutions such as community transport, assistance schemes, volunteer services and adapted taxis.

Useful Contacts & Resources: DVLA Medical Enquiries: 0300 790 6806. DVLA medical information (online): gov.uk/stroke-and-driving. Driver and Vehicle Licensing Agency (DVLA), Swansea: address on STR1 form. Driving assessment centres and Driving Mobility: many regions have specialist driving assessment services. Stroke Association (UK): for general support and information. Regional NHS stroke and rehabilitation services: your local hospital or community team.

Frequently Asked Questions (FAQs): I feel completely “back to normal” before one month is over. Can I drive?: No. The law mandates a minimum no-drive period of one month, regardless of how well you feel.

Do I always have to send in my driving licence to DVLA?: Not necessarily. Usually you only need to provide a medical report and relevant forms such as STR1, and DVLA may issue decisions without you surrendering your licence.

If I had a TIA (mini-stroke), do the same rules apply?: Yes. You must not drive for one month after a TIA. If you recover fully, you may not need to notify DVLA.

What if I had multiple TIAs in a short period?: A three-month no-drive period may be required.

If I have a Group 2 (bus or lorry) licence, are the rules different?: Yes. You must inform DVLA immediately, and you are disqualified from driving for at least one year.

Summary (What You Must Remember): Stop driving straight away after a stroke or TIA. Do not drive for at least one month. After one month, if you have any continuing issues that affect safe driving, notify DVLA. Undergo assessments (physical, vision, cognitive) before returning. DVLA will review and decide whether and how you may drive. Always inform your insurance company. If driving is no longer safe or allowed, explore alternative transport options.

ChatGPT-5 (Thinking) - Standardised Text

Driving After a Stroke (UK):

Patient information for adults with a car or motorcycle licence (Group 1) and bus/lorry licence (Group 2). Last reviewed: 27 September 2025.

The short answer: Do not drive for at least 1 month after a stroke or TIA (mini-stroke). You may drive again after 1 month only if you have made a satisfactory recovery and no problems remain that could affect safe driving. If problems remain at 1 month, tell the DVLA.

Who must tell the DVLA?:

Group 1 (car/motorcycle): Stroke: You must stop driving for 1 month. If any deficits remain after a month, you must notify DVLA and should not drive until DVLA says you can. TIA: Do not drive for 1 month. If you have fully recovered and have no further TIAs, you usually do not need to notify DVLA; do tell your insurer. If symptoms persist beyond a month, notify DVLA.

Group 2 (bus/lorry): Stroke or TIA: You must tell DVLA and stop driving. The typical minimum off-driving period is at least 1 year, with DVLA assessment before any return. Use form STR1V to notify.

Specific issues that can delay return to driving: Seizure soon after stroke (a “provoked” seizure): You must stop driving and notify DVLA. Usual minimum off-driving period is 6 months for Group 1, longer for Group 2. Visual field loss (e.g., hemianopia): Often not compatible with Group 1 standards; DVLA usually requires a binocular Esterman field test. Some “exceptional case” relicensing may be possible in strictly defined circumstances. Do not drive and notify DVLA. Cognitive problems (memory, attention, processing): Notify DVLA; you must be safe to drive.

Getting back on the road-step by step (Group 1): First month: Do not drive. Focus on recovery and rehabilitation. At approximately 4 weeks: Speak to your stroke team or GP about fitness to drive. If no deficits affect driving, you can usually resume. If deficits remain, notify DVLA and do not drive. Insurance: Inform your insurer that you have had a stroke or TIA. Driving without declaring relevant medical conditions may invalidate cover. If in doubt: Ask about a Driving Mobility assessment.

Practical tips: Vision: Book an optician or orthoptic review if you notice new blurring, double vision or field loss. Fatigue and attention: Only return when you can concentrate for sustained periods without excessive fatigue. Adaptations: If you have residual physical limitations, vehicle adaptations and specialist driving tuition may help; the DVLA can consider licences with conditions.

How to notify DVLA: Online guidance: Search “GOV.UK stroke and driving” for the current pages and online forms. Group 2 (bus/lorry): Use form STR1V to report a stroke or TIA.

When should I seek urgent advice?: New symptoms such as weakness, speech or vision problems, any seizure, or another TIA: stop driving and contact your stroke team, 111 or 999 as appropriate. For multiple TIAs over a short period, longer off-driving periods may apply-seek medical advice.

Disclaimer: This leaflet summarises UK rules as of 27 September 2025. DVLA standards can change and individual cases vary. Always follow advice from your stroke team or GP and the DVLA’s latest guidance. If you are unsure, do not drive until you have checked.

Appendix C

Standardisation Protocol (Exact, Deterministic Recipe)

To minimise formatting bias without altering wording, all files (LLM leaflets and comparator) were converted to plain text and normalised using a structure-only pipeline applied identically across documents.

Preserve lexical content. Keep original wording, spelling (UK/US as written), numerals, abbreviations, and word order. No paraphrasing, synonym swaps, recasing, or grammar edits.

Lists into sentences. Remove bullet/hyphen symbols; render one item per line, left-aligned, ending with a full stop; preserve original item order.

Headings/subheadings. Retain wording verbatim. Add a trailing colon to declarative headings that lack punctuation. Ensure interrogative headings end with a question mark (no trailing colon).

Tables into punctuation-only flatten. For each row, join cells as Label: value. (repeated as needed); keep original cell order; do not add connectives (“and/then/which/that”).

References & emojis. Remove inline citations (e.g., "〔12〕", "(Smith 2023)", source markers), reference lists, footnote markers and all emojis/decorative symbols (e.g., ticks/stars).

Non-content artefacts. Remove headers/footers, page numbers and “see page …” cross-references.

Extraction artefacts. Resolve line-break hyphenation (e.g., “manage-\nment” → “management”), delete zero-width/soft-hyphen characters, and collapse multiple spaces to one.

Whitespace & spacing. One blank line between sections; none within paragraphs; single space after punctuation; trim leading/trailing spaces.

Content to retain. Keep end-user contact details (e.g., helplines, service URLs/emails/phone numbers) when part of the leaflet content.

Quality check. Visually compare each standardised file to its source to confirm only the structural operations above were applied.

All readability metrics were computed after this standardisation step.
